# Scientific publications in critical care medicine journals from East Asia: A 10-year survey of the literature

**DOI:** 10.12669/pjms.322.8782

**Published:** 2016

**Authors:** Zhenyu Cao, Chongyang Ou, Hongfei Teng, Xiguang Liu, Hongxin Tang

**Affiliations:** 1 Zhenyu Cao, Department of Critical Care Medicine, NO. 413 Hospital of PLA, NO.98 Wenhua Road, 316000 Zhoushan, China; 2 Chongyang Ou, Department of Medical Affair, NO. 413 Hospital of PLA, Zhoushan, China; 3 Hongfei Teng, Department of Critical Care Medicine, NO. 413 Hospital of PLA, NO.98 Wenhua Road, 316000 Zhoushan, China; 4 Xiguang Liu, Department of Critical Care Medicine, NO. 413 Hospital of PLA, NO.98 Wenhua Road, 316000 Zhoushan, China; 5 Hongxin Tang, Department of Critical Care Medicine, NO. 413 Hospital of PLA, NO.98 Wenhua Road, 316000 Zhoushan, China

**Keywords:** Critical care medicine, China, Japan, South Korea, Bibliometric analysis

## Abstract

**Objective:**

The quantity and quality of publications in critical care medicine from East Asia haven’t been reported. This study aimed to investigate the contribution of publications from East Asia.

**Methods:**

Articles from China, Japan and South Korea in 2005 to 2014 were retrieved from Web of Science and Pubmed. The number of publications, impact factor, citation, and article types were analyzed.

**Results:**

There were 3076 publications from East Asia (1720 from China, 913 from Japan, and 443 from South Korea). There were a significant decrease in publications from Japan (p = 0.024) and significant increases from China (p = 0.000) and South Korea (p = 0.009). From 2006, the number of articles from China exceed Japan. China had the highest total impact factor (6618.48) and citation (18416), followed by Japan (4566.03; 15440) and South Korea (1998.19; 5599). Japan had the highest mean impact factor (5.00) and citations (16.91), followed by South Korea (4.51; 12.64) and China (3.85; 10.71).

**Conclusions:**

China and South Korea`s contributions to critical care medicine had significant increases during the past 10 years, while Japan had a significant decrease. China was the most productive region in East Asia since 2006. Japan had the highest quality research output.

## INTRODUCTION

East Asia is rapidly developing and has achieved great advances in critical care medicine in recent years.^1^-^3^ It is the eastern sub-region of the Asian continent, including Japan, South Korea, North Korea, Mongolia, and China, comprising Mainland (ML), Hong Kong (HK), Taiwan (TW), and Macau.^3^ This is an important region in the world because Japan is one of the leading developed countries; ML with the largest population has the most rapid economic growth. In addition, three of Asia’s four dragons, including HK, TW, and South Korea, are located in East Asia. However, East Asia’ contribution to critical care medicine has not been investigated.

The number of publications by an institution or country is one indicator of its research contribution.^1^ Recently, it has been widely used to investigate East Asia’ contribution to the world body in several medical fields.^4^-^6^ However, to our knowledge, the quantity and quality of East Asian research production in critical care medicine have not been reported. Therefore this study aimed to investigate the contributions of articles from East Asia to critical care medicine during a 10-year period.

## METHODS

### Search strategy

A total of 27 journals were identified from the “critical care medicine” category of the 2013 Journal Citation Reports (JCR).^7^ We only included the journals published in English, and therefore non-English-language journals were excluded. Because this analysis was a 10-year literature survey, the journals that have been cited by JCR only in recent years, not including the full 10 years of our survey, were excluded also. Finally, 21 journals were included, as listed in Table-I.

**Table-I:** Journals included in search from 2005 to 2014.

Journal name	Abbreviation	Impact factor
American Journal of Respiratory and Critical Care Medicine	AJRCCM	11.986
Chest	Che	7.132
Critical Care Medicine	CCM	6.147
Intensive Care Medicine	ICM	5.544
Critical Care	CC	5.035
Journal of Neurotrauma	JN	3.968
Resuscitation	Res	3.960
Current Opinion in Critical Care	COCC	3.183
Seminars in Respiratory and Critical Care Medicine	SRCCM	3.022
Shock	Sho	2.732
Neurocritical Care	NC	2.604
Critical Care Clinics	CCC	2.495
Injury	Inj	2.462
Pediatric Critical Care Medicine	PCCM	2.326
Journal of Critical Care	JCC	2.191
Critical Care and Resuscitation	CCR	2.154
Respiratory Care	RC	1.840
Burns	Bur	1.836
American Journal of Critical Care	AJCC	1.600
Anaesthesia and Intensive Care	AIC	1.470
Critical Care Nurse	CCN	1.074

In April, 2015, literature search was performed using Web of Science and Pubmed. Articles published between 2005 and 2014 in these 21 journals were identified. Only original articles and reviews were included. Letters, editorial material and correction were excluded. The “Reprint Address” of each article was considered as the source region.^8^,^9^ No articles was collected from North Korea, Mongolia, and Macau during the study period, so the articles from Japan, South Korea and China (including ML, HK, and TW) were selected accordingly.

### Data extraction

Two reviewers independently conducted study selection and data extraction. Disagreements were resolved by discussion. The number of publications were used to evaluate the quantity of research output. The quality of research was assessed by impact factor (IF) and citation reports. The total number of articles, the total and mean impact factor, and the total number of citation and mean citation per article were collected. In order to analyze the article types, the number of clinical trials (including cross-sectional and prospective cohort studies), randomized controlled trials (RCTs), meta-analyses and case reports were compiled using the publication type categories of the Pubmed database. In addition, articles published on the high-impact journals (IF>5) were generated, and the five most popular critical care medicine journals of the three countries were also determined according to the number of publications.

### Statistical analysis

Descriptive statistics (eg, total, mean) were mainly used in this study. Regression analysis was used to determine significant changes in time trend between 2005 and 2014. Data analysis was performed using statistical software SPSS version 19.0 (SPSS Inc., Chicago, IL, USA), and p < 0.05 was considered statistically significant.

## RESULTS

### Total number of articles

**Fig.1:**
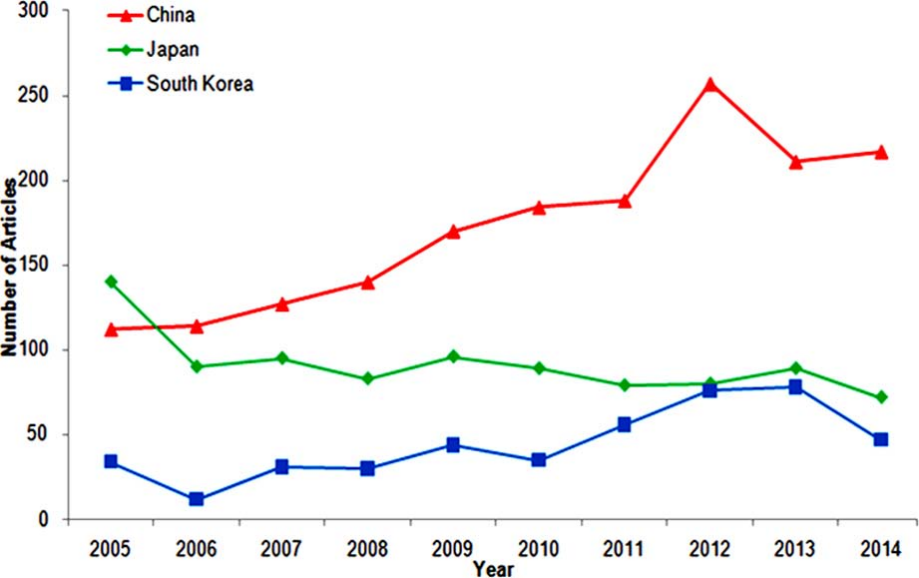
Number of articles from Japan, China, and South Korea in 2005-2014.

A total number of 3076 articles were published from East Asia between 2005 and 2014. China published the most articles (1720/3076; 55.92%) including 913 from ML, 662 from TW and 145 from HK, followed by Japan (913/3076; 29.68%) and South Korea (443/3076; 14.40%). Fig.1 shows the publications from China, Japan, and South Korea. The annual total numbers of articles from the China and South Korea increased significantly from 2005 to 2014 (from 112 to 217, p = 0.000; from 34 to 47, p = 0.009). However, Japan had a significant decrease in the number of yearly publication (from 140 to 72; p = 0.024). From 2006, the number of articles published from China exceeded Japan.

### Impact factor

The total impact factors of articles from China (6618.48) were higher than that from Japan (4566.03) and South Korea (1998.19) (Table-II). However, Japan had the highest mean impact factor of 5.00, followed by South Korea (4.51), and China (3.85) (Table-III). Among the three regions of China, ML had the highest total impact factor (3252.99), while HK had the highest mean impact factor (4.60) (Table-II and Table-III).

**Table-II:** Total impact factor of the articles from Japan, South Korea and China.

Year	Japan	South Korea	China			

			ML	TW	HK	Total
2005	821.34	216.71	127.40	316.06	45.44	488.90
2006	488.88	83.38	95.85	268.73	137.44	502.02
2007	526.59	158.82	166.71	247.58	88.43	502.71
2008	448.51	140.70	184.17	330.26	48.49	562.92
2009	454.76	209.87	299.38	274.42	94.67	668.46
2010	429.48	164.11	328.72	257.99	106.18	692.89
2011	356.04	247.19	396.20	276.53	31.63	704.36
2012	343.91	301.38	591.68	295.08	26.44	913.19
2013	407.68	303.32	498.35	193.68	50.65	742.68
2014	288.84	172.69	564.53	238.69	37.14	840.35

Total	4566.03	1998.19	3252.99	2698.99	666.50	6618.48

**Table-III:** Mean impact factor of the articles from Japan, South Korea and China

Year	Japan	South Korea	China			

			ML	TW	HK	Total
2005	5.87	6.37	3.98	4.79	3.25	4.37
2006	5.43	6.95	3.31	4.55	5.29	4.40
2007	5.54	5.12	3.33	4.20	4.91	3.96
2008	5.40	4.69	3.54	4.23	4.85	4.02
2009	4.74	4.77	3.52	4.29	4.51	3.93
2010	4.83	4.69	3.46	3.74	5.31	3.77
2011	4.51	4.41	3.60	4.01	3.51	3.75
2012	4.30	3.97	3.61	3.60	2.40	3.55
2013	4.58	3.89	3.53	3.23	5.06	3.52
2014	4.01	3.67	3.64	4.26	6.19	3.87

Total	5.00	4.51	3.56	4.08	4.60	3.85

### Citation

The total citation of the published articles from 2005 to 2014 in China (18416) were higher than that in Japan (15440) and South Korea (5599) (Table-IV). However, Japan had the highest mean citation per article (16.91), followed by South Korea (12.64), and China (10.71) (Table-V). Among the three regions of China, ML had the highest total citations (8120), while HK had the highest mean citation per article (16.31) (Table-IV and Table-V).

**Table-IV:** Total citation of the articles from Japan, South Korea and China.

Year	Japan	South Korea	China			

			ML	TW	HK	Total
2005	3985	1186	714	1494	262	2470
2006	2996	388	513	1284	822	2619
2007	2107	717	1061	1008	321	2390
2008	1804	629	1049	1315	128	2492
2009	1550	655	1052	851	314	2217
2010	1174	621	1158	703	339	2200
2011	766	635	987	626	93	1706
2012	590	486	1007	437	20	1464
2013	400	227	438	153	59	650
2014	68	55	141	60	7	208

Total	15440	5599	8120	7931	2365	18416

**Table-V:** Mean citation of the articles from Japan, South Korea and China.

Year	Japan	South Korea	China			

			ML	TW	HK	Total
2005	28.46	34.88	22.31	22.64	18.71	22.05
2006	33.29	32.33	17.69	21.76	31.62	22.97
2007	22.18	23.13	21.22	17.08	17.83	18.82
2008	21.73	20.97	20.17	16.86	12.80	17.80
2009	16.15	14.89	12.38	13.30	14.95	13.04
2010	13.19	17.74	12.19	10.19	16.95	11.96
2011	9.70	11.34	8.97	9.07	10.33	9.07
2012	7.38	6.39	6.14	5.33	1.82	5.70
2013	4.49	2.91	3.11	2.55	5.90	3.08
2014	0.94	1.17	0.91	1.07	1.17	0.96

Total	16.91	12.64	8.89	11.98	16.31	10.71

### Article types

China published the largest number of clinical trials (161), RCTs (99), meta-analyses (41) and case reports (81), as compared with Japan (89 clinical trials, 35 RCTs, 3 meta-analyses and 51 case reports) and South Korea (75 clinical trials, 43 RCTs; 1 meta-analysis, 12 case reports) (Table-VI).

**>Table-VI:** Number of clinical trial, meta-analysis, randomized controlled trial (RCT)and case report from Japan, China, and South Korea in 2005-2014.

Study type	China	Japan	South Korea
Clinical trial	161	89	75
Meta-analysis	41	3	1
Randomized controlled trial	99	35	43
Case report	81	51	12

### High-impact critical care medicine journals

There are five journals with IF > 5 in the 2013 JCR (Table-I). A total of 955 articles published in the five high-impact journals in China, Japan and South Korea between 2005 and 2014 (Table-VII). Among these journals, *Chest* was the journal with the most articles (428/955; 44.82%), followed by *Critical Care Medicine* (230/955; 24.08%) and *Critical Care* (213/955; 22.30%). China published the largest number of articles (487/955; 50.99%) in the five high-impact journals, followed by Japan (343/955; 35.92%) and South Korea (125/955; 13.09%) (Table-VII).

**Table-VII:** Articles published in the high-impact journals from Japan, South Korea and China.

Rank	Journal	Japan	South Korea	China				Total

				ML	TW	HK	Total	
1	AJRCCM	3	1	1	10	3	14	18
2	Chest	190	69	66	64	39	169	428
3	CCM	71	21	49	83	6	138	230
4	ICM	23	5	11	23	4	38	66
5	CC	56	29	89	37	2	128	213

Total		343	125	216	217	54	487	955

### Popular critical care medicine journals

The most popular journals for the East Asian authors in the field of critical care medicine are listed in Table-VIII. *Chest*, *Resuscitation* and *Shock* were the most popular journals in Japan (190), South Korea (73) and China (260), respectively. *Chest* was the only journal appeared in all top five journals in the three regions. *Resuscitation*, *Shock, American Journal of Respiratory and Critical Care Medicine*, *Critical Care Medicine*, and *Injury* were found in the top five journal list in two regions.

**Table-VIII:** The five most popular critical care medicine journals in Japan, South Korea and China.

Rank	Japan	South Korea	China			

			ML	TW	HK	Total
1	Che(190)	Res(73)	Bur(177)	Sho(128)	Che(39)	Sho(260)
2	Sho(133)	Che(69)	Inj(142)	CCM(83)	Inj(20)	Bur(242)
3	Res(94)	JCC(43)	Sho(132)	Inj(73)	Res(17)	Inj(235)
4	AJRCCM(80)	Inj(42)	CC(89)	Res(70)	Bur(16)	Che(169)
5	CCM(71)	AJRCCM(36)	JN(68)	Che(64)	AIC(15)	CCM(138)

## DISCUSSION

East Asia is increasingly important to the world due to its rapid developments in economy, science and technology. However, the status of scientific research on critical care medicine from East Asia have not been investigated. Publication is an important indicator of the advancement of scientific research. It has been widely used to study the contribution of East Asia to the whole world.^4^-^6^ This study gives an insight into the research output from East Asia in the field of critical care medicine.

Some East Asian regions, especially Japan, played an important role in the scientific and medical research in the past decades. However, a significant decrease trend of Japanese scientific publications in the field of critical care medicine was observed in our study. This trend was also found in other medical fields.^4^,^10^,^11^ There may be several reasons. First, the recession of Japanese economy in the past decades might lead to the decrease of financial funding support to medical research and subsequent the decrease of publications.^4^ Second, from 2004, the program of compulsory training on Japanese medical residents might induce the decrease of scientific research power in the medical universities.^10^

The progress of research productivity may mirror the financial status of the countries, especially for China. Growing contribution to scientific research from China has been proved in many biomedical fields.^4^-^6^,^8^,^9^,^11^ It also holds true for the field of critical care medicine. China published the largest number of clinical trial, meta-analysis, RCT and case reports, indicating a comprehensive progress in the field of critical care medicine. There are several possible reasons for this trend. First, the rapid economic development of China may lead to the increasing funding in critical care medicine.^8^,^9^ Second, China has an advantage in recruitment of participants in medical research due to the largest population in the world.^8^,^12^ Third, clinical trials performed in China may be much less expensive than other developed countries.^4^,^12^ Therefore China may give more contribution to critical care medicine in the future.

In this study, articles published by China had the greatest number of total impact factor and total citation. This result suggests that China has the greatest overall impact in critical care medicine in East Asia. One of the main reasons may be that China published the most articles. When mean impact factor and mean citation were used to assess the quality of publication, Japan ranked the highest, indicating that Japan published more high quality research than China and South Korea.

The most popular journal for Japan was *Chest*, the most popular journal for South Korea was *Resuscitation*, and the most popular journal for China was *Shock*. This finding indicate that different countries may have different research power. *Chest* appeared in all the top-5 journals in the three countries. It suggests the important influence of this journal in East Asia.

There are some limitations in this study. First, only impact factor and citation were used to evaluate the papers in this study, despite there were many measurements, such as Scimago and H index. Second, the journals were selected from the critical care medicine category of the JCR, but some journals in general medicine journals, such as some high ranked journals, also published some articles related to critical care medicine. Nevertheless, the journals included in this survey represent the major international journals devoted to the discipline of critical care medicine.

## CONCLUSION

The number of articles increased significantly in both China and South Korea during the past 10 years, while decreased significantly in Japan. From 2006, China was the most productive region in East Asia. Japan had the highest quality research output according to mean citation and mean impact factor per article.
